# Advantages of Extracellular Ubiquitin in Modulation of Immune Responses

**DOI:** 10.1155/2016/4190390

**Published:** 2016-08-25

**Authors:** Rusudan Sujashvili

**Affiliations:** Department of Biophysics, I.Beritashvili Center of Experimental Biomedicine, 14 Gotua, 0160 Tbilisi, Georgia

## Abstract

T and B lymphocytes play a central role in protecting the human body from infectious pathogens but occasionally they can escape immune tolerance, become activated, and induce autoimmune diseases. All deregulated cellular processes are associated with improper functioning of the ubiquitin-proteasome system (UPS) in eukaryotic cells. The role of ubiquitin in regulation of immune responses and in autoimmune diseases is only beginning to emerge. Ubiquitin is found in intra- and extracellular fluids and is involved in regulation of numerous cellular processes. Extracellular ubiquitin ascribed a role in lymphocyte differentiation. It regulates differentiation and maturation of hematopoietic cell lines. Ubiquitination is involved in initiation, propagation, and termination of immune responses. Disrupted ubiquitination can lead to autoimmunity. Recent observations showed that it can suppress immune response and prevent inflammation. Exogenous ubiquitin may provide good potential as a new tool for targeted therapy for immune mediated disorders of various etiologies.

## 1. Introduction

Synthesis and degradation of proteins is constant process in all cells. Protein content is regulated by the cycle of its permanent turnover and is crucial for cell survival. Derangements of protein metabolism lead to the development of severe diseases and mainly are caused by diverse mechanisms. Different factors can cause ubiquitin system dysfunction and contribute to the accumulation of damaged proteins. UPS plays an important role in regulation of cellular processes via degradation of damaged proteins and control of protein quantity in the eukaryotic cell. Almost all cellular processes including antigen processing, immune response and inflammation, and modulation of cell surface receptors are under control of UPS [1]. It is suggested that extracellular ubiquitin is an immune modulator affecting T and B lymphocytes [2]. Extracellular ubiquitin has important implications to investigation of pathways involved in regulation of immune system. Ubiquitin appears to be a modulator of innate and adaptive immune responses. Ubiquitination regulates initiation, propagation, and termination of immune responses.

## 2. UPS

Ubiquitin is a small regulatory highly conserved protein and occurs in all eukaryotic cells. The attachment of ubiquitin to the *ε*-amine lysine residues of target proteins in course of mono- and polyubiquitination requires a series of ATP-dependent enzymatic steps by ubiquitin activating enzyme (E1), ubiquitin conjugating enzyme (E2), and ubiquitin ligase (E3). The C-terminal Gly76 residue of ubiquitin is a key residue that functions in diverse chemistry of ubiquitin reactions. Ubiquitin can be conjugated to itself via one of the seven lysines sequenced in ubiquitin molecule (K6, K11, K27, K29, K33, K48, and K63) by forming covalent isopeptide bond. Cell contains numerous E3 enzymes that recognize particular target proteins by structural features and thus account for substrate selectivity [3]. Ubiquitination can regulate the activity and location of target proteins by means of monoubiquitination or alternate multiubiquitination. E3 ligases are represented as members of two different families. Protein C-terminus domains found in E3, homologous to the E6-AP carboxyl terminus (HECT), act as monomers and ubiquitin is initially transferred to a cysteine residue of a ligase before it is transferred to substrate. The E3 containing zinc cations binding motif (RING/U-box protein) family members function as a scaffold that binds to both an E2 bound to ubiquitin and a substrate. Proteasomes recognize polyubiquitin tetramers linked through lysin-48 as a signal to deubiquitinate and destroy the substrate. Finely, free and reusable ubiquitin and amino acids are released by the action of a large variety of ubiquitin C-terminal hydrolases and ATP-ases [4, 5]. Recently a new type of polyubiquitin linear chain has been identified in which the C-terminal glycine of ubiquitin is conjugated to the *α*-amino group of the amino-terminal methionine of another ubiquitin [6]. UPS is the major intracellular degradation pathway and the defects in clearance of cellular proteins can lead to autoimmunity.

## 3. K Linkages Involved in Immune Modulation

Polyubiquitin chains formed via various linkages carry distinct structural and functional information. Ubiquitin chains linked by different lysine residues define the location and character of protein processing. K48 ubiquitin chains target substrates to the proteasome for degradation. In contrast, Lys63- or K6-linked chains perform nonproteolytic functions in at least four pathways: DNA damage repair, endocytosis, cellular signaling, intracellular trafficking, and ribosomal biogenesis [7, 8]. Polyubiquitin chains linked through K63 and K48 participate in innate immune responses via activation of pattern recognition receptor, proteins expressed by the cells of innate immune system (PRRs) that will result in activation of nuclear factor kappa-B (NF-kB) and the following induction of cytokines: tumor necrosis factor (TNF) and interleukin-1 (IL-1) [9]. K33-linked polyubiquitination of T cell receptors (TCR-Z) functionally modifies the receptor phosphorylation and protein binding in a proteolysis independent manner. K11 linkage mediates proteasomal degradation in mitosis, cell cycle regulation, membrane protein trafficking, and TNF signaling [10]. Alternatively, monoubiquitination can lead to export and translocation of proteins into the cytoplasm. Linear polyubiquitin chains are involved in termination of TNF-induced cell death. Mutated ubiquitin-like-domain-containing complex (SHARPIN), component of linear ubiquitin chain assembly complex (LUBAK), causes immune system disorders and multiorgan inflammation. SHARPIN deficiency in mice causes activation of inhibitor of nuclear complex kappa-B kinase (IKK) and NF-kB in B cells, macrophages, and embryonic fibroblasts. It leads to rapid cell death upon TNF-alpha stimulation via caspase-8 dependent manner [11]. In eukaryotic cells several ubiquitin binding proteins (UBPs) are determined to be essential for immune signaling pathways [12, 13].

## 4. E3 Ligases and Deubiquitinases (DUBs) as Potential Targets for Immune Therapy

Ubiquitin adding (i.e., ubiquitylation, a reversible in vivo covalent modification of target proteins) affects antigen processing by antigen-presenting cells and upgrades immunological tolerance by modification signaling components to move the balance away from activation and toward anergy. Decreased expression of E3 ligases induces autoimmunity by the loss of immune tolerance and the absence of epitopes against the immune system [14]. Dysfunction of E3 ligases, which catalyze the final step of ubiquitin attachment, can lead to indiscriminate T cell activation and loss of tolerance to self-antigens [15]. The induction of anergy in T cells is an active process that is dependent on new protein synthesis and is associated with the increased expression of E3 ubiquitin ligases Cbl-b, Itch, and GRAIL and other negative regulators of TCR signaling [16–23].

T cell activation is subject to tight regulation to avoid inappropriate responses to self-antigens. In mice lacking both Itch and Cbl-b E3 ubiquitin protein ligases that cooperate in K33-TCR-Z linkage spontaneously develop systemic autoimmunity characterized by splenomegaly, lymphocyte infiltration of lungs, liver, kidneys, and heart correlating with increased TCR-Z phosphorylation and accumulation of cytokines in serum [24]. In the absence of Cbl-b, T cells are hyperproliferative and are able to be fully activated even without CD28 costimulation [25–28]. As a consequence, Cbl-b total body knockout mice develop spontaneous autoimmunity resulting in infiltration of T and B lymphocytes in different organs and parenchymal damage [27, 29, 30]. Moreover, the lack of Cbl-b prevents T cell tolerance induction in vivo [2]. Besides Cbl-b, other E3 ligases, such as the HECT-type E3 ligase Itch and the gene related to anergy in lymphocytes RING-type E3 ligase (GRAIL), have been identified as critical regulators of T cell activation and tolerance. As in the case of Cbl-b, Itch and GRAIL deficient T cells hyperrespond to TCR stimulation [31–33] and removal of either of these E3 ligases causes T cells resistant to anergy induction, both in vitro and in vivo [34].

Itch negatively regulates T cell growth factor production and proliferation. Itch attaches ubiquitin to substrate proteins. Ubiquitination of the T cell receptor mediates its downregulation [35]. Human Itch deficiency results in a complex phenotype that affects physical growth, craniofacial morphology, muscle development, and immune function. The consequences of Itch deficiency in humans appear to be similar to those in *Itch* ^−/−^ mice. This is probably a result of immune deregulation in patients with autoimmune disease [36–38].

Genetic deficiency in the pellino E3 ubiquitin protein ligase 1 (Peli1) causes hyperactivation of T cells and increased T cells resistance to suppression by regulatory T cells. Peli1-deficient mice spontaneously developed autoimmunity characterized by multiorgan inflammation and autoantibody production. Peli1 is identified as a critical factor in the maintenance of peripheral T cell tolerance [39].

DUBs the enzymes that disassemble ubiquitin chains and remove ubiquitin from ubiquitin protein conjugates are central to the UPS. DUBs participate in termination of immune responses which is as important as initiation of the process. Ubiquitin carboxyl-terminal hydrolase 15 (USP15) is a negative regulator of T cell activation. DUB USP15 deficiency promotes T cell activation [40]. Conditional knockout of DUB TNFAIP3-tumor necrosis factor, alpha-induced protein 3 (A20) developed autoimmunity. A20 has been reported as a disease susceptibility gene for human inflammatory and autoimmune pathology, including Rheumatoid Arthritis (RA) and Juvenile Idiopathic Arthritis, Systemic Lupus Erythematosus (SLE), Inflammatory Bowel Disease (IBD), celiac disease, psoriasis, type 1 diabetes, Sjogren's syndrome, coronary artery disease, rheumatic heart disease, and systemic sclerosis. As ubiquitin is highly conserved protein it lacks immunogenicity, but ubiquitinated proteins accumulated in cells are highly immunogenic in autoimmune patients [41–43]. The diversity of E3 ligases and DUBs involved in controlling autoimmunity makes these proteins potential targets for immune therapy.

## 5. Extracellular Ubiquitin Is a Promising Regulator of Immune System

Extracellular ubiquitin is found at nanomolar concentrations in human plasma and serum. It is detectable in cerebrospinal fluid (CSF) and bronchoalveolar lavage fluid (BALF). Elevation of plasma ubiquitin levels is found during hairy cell leukemia (HCL), allergic, autoimmune infections, and other disorders [44, 45].

Release mechanisms of ubiquitin into extracellular fluids are not known. Source of extracellular ubiquitin might be the passive release from cells during physiological processes, like apoptosis and necrosis, but some authors report on extraction of ubiquitin from normal cells [46, 47]. Up to date many aspects of extracellular ubiquitin activity remain unclear, specially concerning its possible pathways and the role in various cellular processes involved in immune responses [48].

Injected proteins can be used as authentic tests of the action of endogenous proteins. Extracellular ubiquitin is easily available for protein modifications. It has been used in numerous experiments for elucidation of the role and pathways of extracellular and exogenous ubiquitin. Initially, in vivo injected extracellular ubiquitin was identified as an inducer of both T and B cell differentiation markers on precursors of mouse lymphoid cells. Ubiquitin is capable of inducing the functional differentiation of granulocytes [49]. Extracellular ubiquitin secreted by activated T-lymphocytes was shown to inhibit cytotoxic activity of platelets [50]. Later it was studied as a modulator of hematopoietic progenitor cells. Ubiquitin secreted from hairy cells had an inhibitory effect on the growth of normal hematopoietic progenitor cells [51].

Microinjected extracellular ubiquitin incorporated into hematopoietic cells mediates their growth suppression and apoptosis through proteasome-dependent degradation of selective cellular proteins such as signal transducer and activator of transcription 3 (STAT3) in IL-6-dependent human T-lymphoma cell line (KT-3 cells) [52]. STAT3 is the major mediator of glycoprotein 130 (gp130) subunit of cytokine receptor signal. Inside the cell gp130 is shown to be modified by K63 polyubiquitin chains and directed to lysosome for degradation which is essential for termination of IL-6 signaling [53]. IL-6 mainly functions in hematopoietic and lymphoid cell systems. It is originally identified as a B cell differentiation factor that induces final maturation of B cells. It also acts on T cells and hematopoietic progenitor cells [54].

STAT3 and cytokines are main targets for ubiquitin in process of regulation of hematopoietic cells proliferative activity. Moreover, forkhead box protein 3 is regulated by the E3 ubiquitin protein ligases Itch and Cbl-b and induces regulatory activity of T cells [55]. Itch E3 ubiquitin protein ligase plays a role in lymphoid cell differentiation and regulation of immune responses. Mutation of this protein causes multisystem autoimmune disease [56]. Cbl-b functions as a negative regulator of T cells. It is involved in the regulation of peripheral tolerance and anergy of T cells. Ex vivo generated human monocyte-derived suppressive cells (HuMoSCs) were suggested as inhibitors of effector T-lymphocytes and promoters for expansion of immunosuppressive forkhead box protein 3-positive CD8^+^ regulatory T-lymphocytes. Therefore, they are supposed to be an efficient therapeutic tool to prevent graft-versus-host disease (GvHD) transplant rejection and autoimmune diseases. Interaction of HuMoSCs with T cells is dependent on STAT3 and cytokine activity [57]. Therefore, these data prove the significance of ubiquitination in the abovementioned processes. One can speculate that extracellular ubiquitin might play a significant role in process of HuMoSCs interaction with T Reg cells. Study of extracellular ubiquitin effect on HuMoSCs seems to be an attractive goal for farther investigation of molecular pathways that might play pivotal role in GvHD transplant rejection and autoimmune diseases therapy.

Extracellular ubiquitin was identified as an endogenous agonist of CXC chemokine receptor type 4 (CXCR4) [58]. CXCR4 is a 7-transmembrane G protein-coupled receptor that is expressed by a variety of cells, including peripheral blood lymphocytes, monocytes, thymocytes, and pre-B cells. CXCR4 serves as a receptor for T cell tropic human immunodeficiency virus type 1 (HIV-1) strains. Extracellular ubiquitin binds CXCR4 receptor on monocyte, B cell, and T cell surfaces and induces calcium ions influx into the cells. Affinity of extracellular ubiquitin to B- and monocyte cell surface is higher than to T cell's surface [59]. CXCR4 agonist properties of extracellular ubiquitin indicate its possible role in leukocyte differentiation and in normal and pathological hematogenesis [2]. Extracellular ubiquitin changes the ratios of the heterogeneous population of bone marrow and peripheral blood [60]. In vivo injected exogenous ubiquitin inhibits mitotic activity of bone marrow cells by about 53% in intact rats [61]. Recent observations showed that extracellular ubiquitin can suppress immune response and prevent inflammation [62]. Ubiquitin has been suggested as a promising anti-inflammatory protein therapeutic. Ubiquitin is involved in regulation of immunodepression in critically ill patients. TNF-alpha is associated with increased risk of sepsis in critically ill patients [59]. In vivo induced ubiquitin reduced lipopolysaccharide (LPS) stimulated TNF-*α* production of whole blood and peripheral blood mononuclear cells (PBMNCs). Extracellular ubiquitin incorporated into monocytic cells restores endogenous ubiquitin pool and provides ubiquitin protein ligase system with additional substrates to maintain intracellular protein turnover during immunological responses [62]. Peptide fragment corresponding to the ubiquitin (50–59) sequence possessed the immunosuppressive activity comparable with that of ubiquitin [63, 64].

Ubiquitination of proteins is essential for proper course of normal and pathological cellular processes. However, we are far from fully understanding the multiple pathways of cellular and extracellular ubiquitin involved in regulation and deregulation of numerous cellular activities involved in immune responses. Investigation of the role of extracellular ubiquitin in modulation of immune responses by means of exogenous ubiquitin seems to be informative for this purpose.

## 6. Conclusion

Regulated protein turnover by the UPS is essential for the survival of eukaryotic cells. Alterations in the UPS are demonstrated to be correlated with a variety of human pathologies including autoimmunity, immunodeficiency, hematopoietic, and malignant [48]. The finding that proliferating cells are more sensitive to defects in protein degradation suggests that further emphasis on UPS could provide new therapeutic tools to target disorders in hemato- and lymphogenesis. Extracellular ubiquitin is considered as a disease biomarker, as numerous diseases are associated with increased concentrations of ubiquitin in body fluids [2, 44, 45]. Modification of proteins by ubiquitin may impact their visibility by the immune system; this should highlight the therapeutic potential of manipulating extracellular ubiquitin in autoimmune diseases. Further investigation of extracellular ubiquitin might reveal new pathways involved in development of autoimmunity and other immunological diseases and open new strategies to targeted therapy for immune mediated disorders of various etiologies.
